# Infective Endocarditis After TAVR—Surgical Challenges and Outcomes

**DOI:** 10.3390/jcm14217859

**Published:** 2025-11-05

**Authors:** Andrea Reiter, Julia Schreyer, Melchior Burri, Hendrik Ruge, Markus Krane, Nazan Puluca

**Affiliations:** 1 Department of Cardiovascular Surgery, Institute Insure, German Heart Center Munich, School of Medicine & Health, Technical University of Munich, 80636 Munich, Germanyruge@dhm.mhn.de (H.R.); 2 DZHK (German Center for Cardiovascular Research)—Partner Site Munich Heart Alliance, 80636 Munich, Germany

**Keywords:** transcatheter aortic valve implantation, TAVR, THV, surgical explantation, infective endocarditis

## Abstract

**Background:** Infective endocarditis (IE) after transcatheter aortic valve replacement (TAVR) is a severe complication. Surgical explantation of infected transcatheter heart valves (THV) is technically demanding and associated with high mortality. Data on risk factors for perioperative death and long-term outcomes remain limited. **Aim:** To identify predictors of mortality in patients undergoing surgical aortic valve replacement (SAVR) for IE after TAVR. **Methods:** We conducted a case–control study of patients treated with SAVR for IE after TAVR at our center between February 2008 and December 2023. Fifteen patients who died in hospital (cases) were compared with 35 perioperative survivors (controls). Hospital survivors were followed for long-term outcomes. **Results:** Age, sex, comorbidities (kidney disease, cerebrovascular disease, COPD, diabetes, peripheral artery disease), and anthropometrics were similar between groups. Cases had significantly lower left ventricular function and higher logistic EuroSCORE and STS-PROM before surgery. Causative microorganisms, cross-clamp time, and concomitant procedures did not differ. Postoperative pacemaker implantation, rethoracotomy, stroke, and ICU or hospital stay were comparable, while dialysis was more frequent in cases (44% vs. 25.7%). Median follow-up was 294 days (range 1–3802). Survival was 79.8% at 30 days and 67.4% at 1 year. Of 35 hospital survivors, 29 were discharged home, 6 to rehabilitation/other hospitals; 31 remain alive (1 early, 3 late deaths). **Conclusions:** SAVR for IE after TAVR carries high early mortality (18.1% at 30 days; 32.6% at 1 year). Higher preoperative risk scores and postoperative dialysis were associated with perioperative death. Long-term survival among hospital survivors is favorable, with most patients regaining independent living.

## 1. Introduction

Transcatheter aortic valve replacement has become a well-established procedure and is by now standard of care in elderly patients, including low- to high surgical risk profiles [1,2]. Undeniably, there has been a massive worldwide increase in TAVR procedures over the last decade, especially over the most recent years, with promising clinical outcome data in patients with lower risk [3,4,5].

The increase in TAVR numbers and the expansion of indications to low risk patients mean that complications such as infective endocarditis (IE) will increase in the future. With an incidence of 0.3 to 2.0 per 100 person years, IE after TAVR is rare, but due to significant clinical consequences, it must be taken seriously.

Endocarditis in TAVR patients combines two main issues: prosthetic valve endocarditis (PVE), also has high complication rates with a 1-year mortality rate up to 75% [6,7,8]. Additionally, TAVR patients present further challenges due to their older age and greater number of comorbidities. This results in even higher in-hospital and 1 year mortality rates [9]. The 2023 European Society of Cardiology (ESC) Guidelines on the management of IE emphasize the critical role of multimodality imaging for diagnosis, the importance of Endocarditis Teams, and the need for timely surgery in appropriate patients, even in the presence of neurological complications. In parallel, the 2025 ESC/EACTS Guidelines on valvular heart disease (VHD) highlight the growing role of Heart Valve Centers, advanced imaging modalities, and the Heart Team in guiding treatment strategies for patients with complex valve disease, including those after TAVR. These guidelines expand the recommendations for transcatheter interventions and refine patient selection criteria, while reaffirming that surgical replacement remains necessary in scenarios such as prosthetic valve endocarditis [10,11]. This study aims to contribute to more informed decision making regarding the indication for surgical treatment in this unique patient population. The objective extends beyond evaluating immediate clinical outcomes such as mortality. It also encompasses a broader perspective—assessing the condition in which patients are discharged from the hospital and their ability to return to an independent life at home.

## 2. Methods

### 2.1. Study Design und Study Population, Definitions

We enrolled all consecutive patients who underwent surgical explantation of a transcatheter aortic valve due to infective endocarditis at our institution between February 2008 and December 2023. Patients managed conservatively were excluded since those were not transferred to our surgical department, which may introduce selection bias. All patients were treated according to institutional endocarditis protocols, targeted antibiotic therapy guided by culture or PCR, and individualized surgical planning. In total, 50 patients were analyzed in a case–control study. Mortality and onset of IE were reported according to Valve Academic Research Consortium (VARC)-3 criteria [12].

The STS predicted risk of mortality (STS PROM) and EuroScore were calculated prior to TAVR and SAVR to predict postoperative mortality and to stratify patients into high, intermediate, and low risk.

With the Society of Thoracic Surgeon (STS)-PROM, low risk was defined with a predicted mortality <4%, intermediate risk was defined with a predicted mortality between 4 and 8% and high risk with a predicted mortality >8% [1].

With the logistic European System for Cardiac Operative Risk Evaluation Score, low risk was defined with a predicted mortality <10% and high risk was defined with a predicted mortality ≥10% [13].

Aim of this study was to explore periprocedural mortality. Our goal was to determine parameters associated with death in surgically treated TAVR-IE patients as well a long-term outcome of patients that were discharged. Patients who died in the perioperative period (cases group) were compared to patients that were discharged after SAVR (control group).

### 2.2. Cases Group

Patients who died in hospital after TAVR explantation for IE.

### 2.3. Control Group

Patients who were successfully discharged after TAVR explantation for IE.

### 2.4. Study Aims

The primary aim was to analyze survival at 30 days and 1 year after THV explantation for IE. Our second objective was to determine differences in baseline characteristics between hospital survivors and patients with in-hospital deaths, as well as to further determine long term survival estimates of hospital survivors after the procedure.

We also reviewed data on differences between self-expandable (SEV) and balloon-expandable (BEV) valves, microbiological profiles. Additionally, we analyzed the subsequent transitions of care, particularly the settings to which patients were transferred following their hospital stay.

### 2.5. Statistical Analysis

Categorical variables are reported as counts and frequencies and compared using Fisher’s exact test. Continuous variables are shown as mean (±SD) or median (range) and compared using *t*-tests or Wilcox tests, where appropriate.

Survival rates were depicted using the Kaplan–Meier method and the log-rank test with corresponding 95% confidence intervals (CI). A *p*-value of <0.05 was considered statistically significant. The statistical analyses were performed using SPSS 28.0 (IBM-SPSS Inc., Armonk, NY, USA) and R environment version 4.2.1. (R Core Team (2024). R: A Language and Environment for Statistical Computing. R Foundation for Statistical Computing, Vienna, Austria. URL https://www.R-project.org/) accessed on 16 July 2025.

For the case-control study, unconditional logistic regression was performed to assess the association between surgery for IE and the outcome. Crude and adjusted odds ratios (ORs) with 95% confidence intervals (CIs) were calculated.

### 2.6. Ethical Statement

We obtained local institutional review board approval (ethics committee name: Ethics Committee of the Technical University of Munich, study number 2025-79-S-CB, approval date: 20 May 2025).

## 3. Results

### 3.1. Patient Characteristics/Population

Between February 2008 and December 2023, 93 patients had TAVR explantation in our institution. Of those, in 50 patients the device was explanted due to infectious endocarditis (IE). 15 patients died during the periprocedural stage (case group), while 35 patients survived (control group).

**Figure 1:**
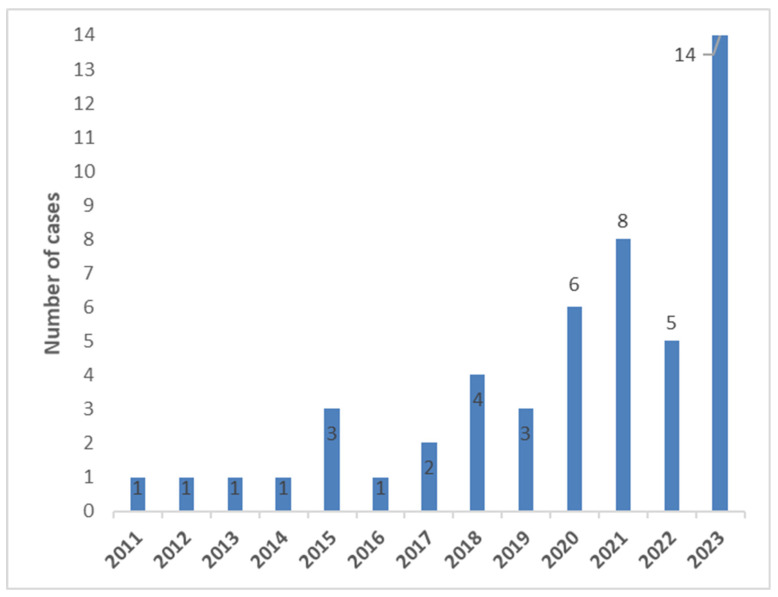
Number of TAVR explant cases (IE) per year.

Number of TAVR-explants for IE per year are shown in Figure 1.

Overall, median age at time of surgery was 78 years [range: 37–88 years]. Median age was 80 years [range: 37–86 years] in the cases group (*p* = 0.8) and 77 years [range: 57–88 years] in the control group.

Patients in the cases group had a significantly lower left-ventricular ejection fraction than patients in control group (42.7 ± 8.4% vs. 49.4 ± 10.6%, *p* = 0.024).

Time between TAVR and SAVR was less than 90 days in 18% of patients (9/50), in 34% (17/50), time between TAVR and SAVR was between 90 days and 1 year, and in 48% (24/50), the TAVR device was explanted beyond a year.

Median time between hospitalization for IE and surgery was 9 days [range: 0–210 days] (data available in 46/50 cases).

Main indications for referral to surgery were valvular/paravalvular extensions, abscess formation, uncontrolled infection, septic emboli, or other causes.

There was no significant difference in age, sex, height, weight, kidney function, history of cerebrovascular disease, COPD, diabetes mellitus, or peripheral artery disease between groups at the time of TAVR explantation. Baseline characteristics are presented in Table 1.

Prior to the initial TAVR, median log EuroScore was 9.5% [range: 1.3–48.3%] (Table 1).

Before SAVR, the median logEuroScore was 28.5% [range: 6.8–82.1%]. Between the cases group and the control group, there was a significant difference (*p* < 0.001) with a median log EuroScore of 53.3% [20.1–82.1%] in the cases group and 26.6% [6.8–64.5%] in the control group.

Median STS-PROM before SAVR was 6.5% [range: 1.3–67.6%] with a median STS-Score of 14.4% [3.9–67.6%] in the cases group and 4.8% [1.3–51.2%] in the control group. There was a significant difference between both groups (*p* = 0.004).

STS-PROM classified patients in three risk groups: 15/50 patients were deemed “low surgical risk”, 14/50 patients were deemed “intermediate surgical risk” and 21/50 patients were deemed “high surgical risk”. There was a significant difference in mortality between these groups (*p* = 0.035).

**Figure 2:**
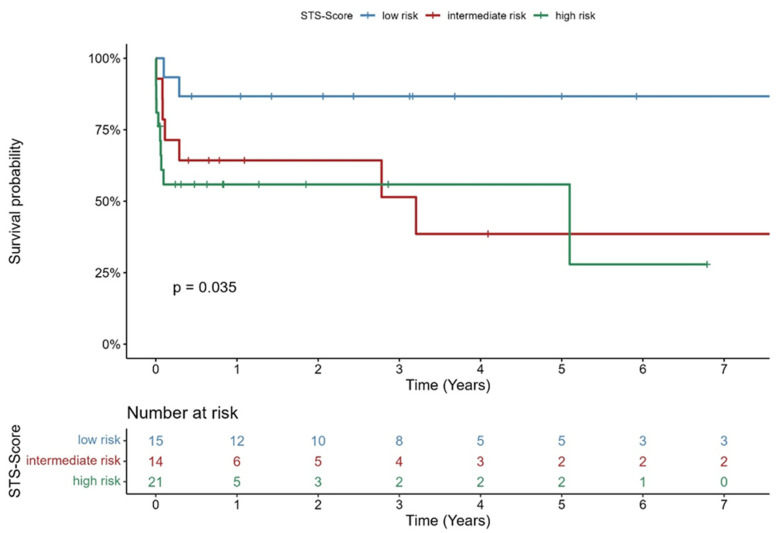
Kaplan–Meier–Survival curve stratified by STS Prom.

The 30 day and 1 year survival in STS-low risk, STS-intermediate risk, and STS-high risk patients was 100% and 86.7%, 92.9% and 64.3%, and 61% and 55.9%, respectively. Kaplan–Meier curve of survival of STS-low-risk, STS-intermediate risk, and STS-high-risk patients is shown in Figure 2.

### 3.2. Causative Microorganism

There was a variety of microorganisms causing IE: coagulase-negative staphylococci was the most common cause (13/50), followed by Enterococci (11/50), Staphylococcus aureus and Streptococcus viridans (8/50, respectively), and others (6/50). In three cases, no causative microorganism could be found. All but one patient were under antibiotic therapy before surgery, either with calculated therapy in cases where blood culture results were not available (yet) or inconclusive or test-appropriate therapy if a causative microorganism was known. The median time of antibiotic therapy prior to surgery was 7 days [range: 1–47 days] (data was not available in 11/50 cases).

Data of causative microorganisms are presented in Table 2.

Polymerase chain reaction (PCR) results after TAVR explantation were available in 42 patients (negative 20/42, positive 22/42).

### 3.3. Periprocedural Characteristics

At surgery, all patients underwent median (re-)sternotomy and SAVR using a bioprosthetic valve.

Explanted TAVR prostheses were mainly BEV (39/50) and did not differ between groups (*p* = 0.12). Median cross-clamp time was 95 min [range: 54 min–348 min], with a median cross-clamp time of 89 min [range: 36 min–233 min] in the cases group and 97 min [range: 52 min–220 min] in the control group (*p* = 0.9).

**Figure 3:**
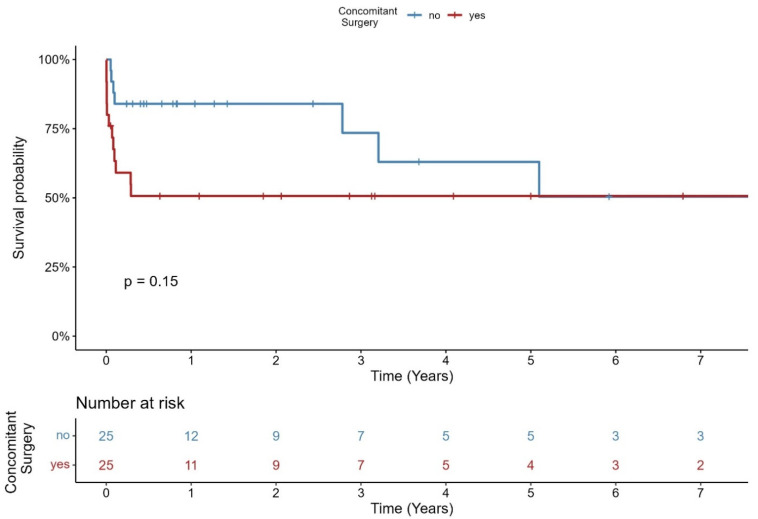
Kaplan–Meier–Survival curve stratified by concomitant surgery.

Concomitant surgery was performed in 50% of all patients (25/50). The Kaplan–Meier–Survival curve of all patients regarding concomitant surgery is shown in Figure 3. In the cases group, 11/15 patients (73.3%) and in the control group 2, 14/35 patients (40%) had concomitant surgery (*p* = 0.062). The most common concomitant procedure was mitral valve surgery (14/50), followed by CABG or CABG plus mitral valve surgery (3/50) and others (8/50).

Periprocedural data is shown in Table 3.

### 3.4. Inhospital Clinical Outcome

**Figure 4:**
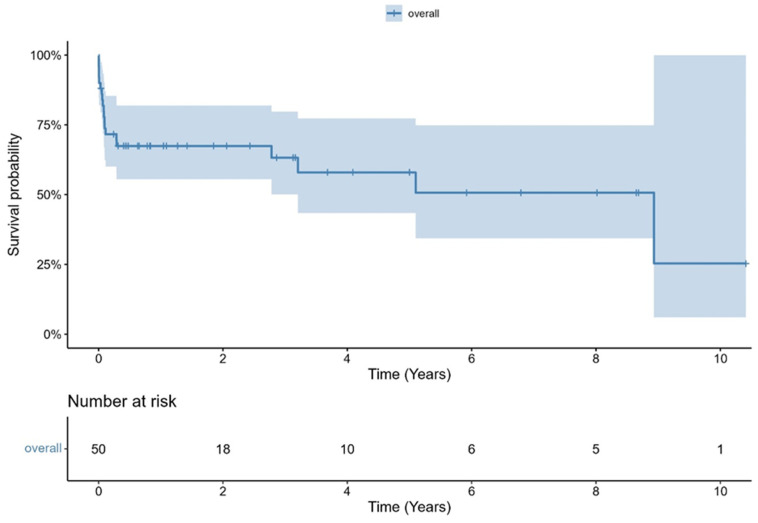
Kaplan–Meier curve of survival.

Median in-hospital length of stay was 20 days [range: 2–95 days]. Thirty days and one year survival was 81.9% and 67.4%, respectively. The Kaplan–Meier curve of overall survival is shown in Figure 4. Data of in-hospital clinical outcomes are presented in Table 4. 15 patients died in the periprocedural stage (cases group). Two patients died of non-cardiovascular causes, and 13 patients died of cardiovascular causes. Median time between SAVR and death in the cases group was 22 days [range: 1–106 days].

### 3.5. Follow Up Beyond Discharge

Follow up was 100% with a median follow-up of 22 days [range: 1–106 days] in the cases group and 752 days [range: 19–3802 days] in the control group.

**Figure 5:**
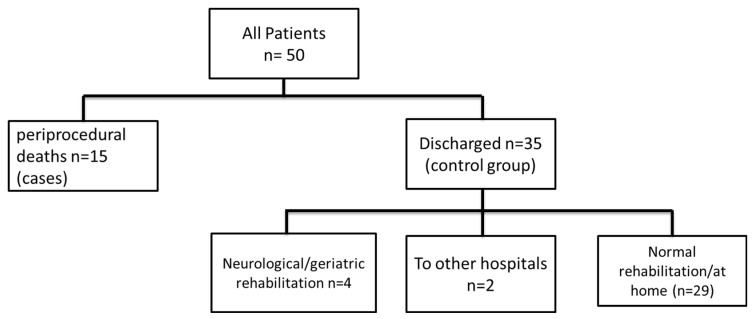
Flow chart of patient discharge target.

There are 35/50 patients who were alive and discharged (=control group). Of those 35 patients, 4 patients were discharged to neurological or geriatric rehabilitation, 2 patients were discharged to other hospitals, and 29 patients were discharged home: either prior to rehabilitation and consecutive home-based life or after in-hospital recovery. Of those 29 patients that went home, 4 patients died subsequently (1 early death, 3 late deaths). A flowchart with a summary of patients discharge targets is shown in Figure 5.

### 3.6. Early and Late IE

Overall, median time between TAVR and TAVR explantation was 490 days [range: 35–1634 days] in the cases group and 328 days [range: 74–3248 days] in the control group (*p* = 0.6).

18% (n = 9) of patients had TAVR explantation within 90 days after TAVR (n = 4; 33% in the cases group and n = 5; 11% in control group); 17% of patients (n = 34%) had TAVR explantation between 90 days and 1 year (13.3% in cases group (n = 2) and 42.9% in control group (n = 15)). In total, 48% of all patients (n = 24) had TAVR explant beyond 1 year after TAVR (53.3% in cases group (n = 8) and 45.7% in control group (n = 16)), which was not significant between groups (*p* = 0.07). Data is shown in Table 1.

**Figure 6:**
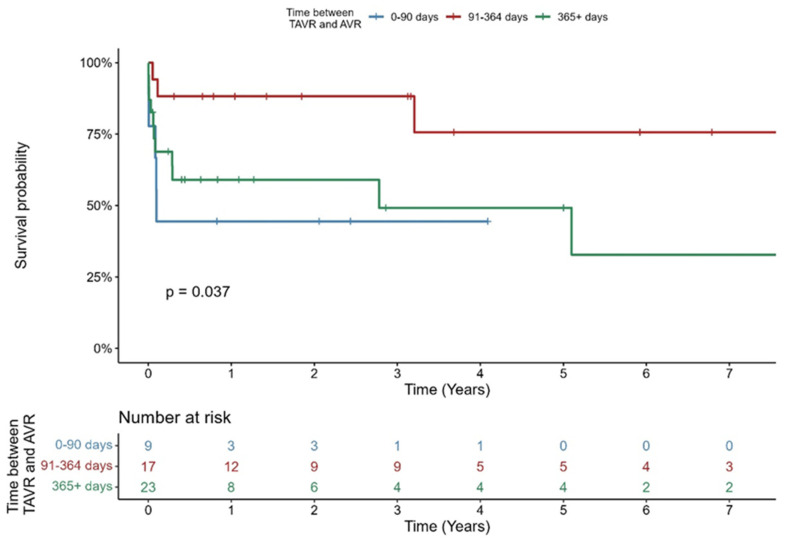
Kaplan–Meier-Survival curve stratified by timing of IE.

Kaplan–Meier estimated survival of all patients categorized in three timeframes after TAVR until TAVR explant is shown in Figure 6. Survival at 30 days and 1 year was 77.8% and 44.4% for patients within 90 days between TAVR and AVR, 94.1% and 88.2% for patients with 90 days to 1 year between TAVR and SAVR, and 73.4% and 59% for patients with more than 1 year between TAVR and SAVR. There was a significant difference between groups (*p* = 0.037).

## 4. Discussion

The main findings of our study are as follows:

(1) Overall survival was 81.9% at 30 days and 67.4% at one year.
(2) Patients with low STS-PROM at the time of TAVR explantation had better survival compared to those with intermediate or high operative risk.
(3) Early mortality was associated with significantly higher logistic EuroScore and STS-PROM, and higher rates of postoperative renal failure, but not with causative microorganisms, cross-clamp time, or concomitant surgeries.
(4) Most surviving patients recovered well and returned to independent living.

### 4.1. Mortality

Comparisons regarding mortality after TAVR explant are difficult, as most studies have small sample sizes and patient selection is inhomogeneous. Studies focus mainly on TAVR-associated infective endocarditis (IE), while details on surgical therapy are rather secondary.

There are reports on a consecutive increase in patients with TAVR IE [14,15]. We confirm these findings: two-thirds of our patients have undergone surgery in the past four years, and only one-third of patients in the nine years before that.

In addition, while absolute numbers are on the rise, Caceres et al. report a decrease in prosthetic valve endocarditis (PVE) operative mortality over time from 22.5% to 10.4% [16].

Guidelines recommend surgery in patients with IE in presence of complications such as uncontrolled infection, extension into paravalvular tissue, emboli, severe prosthetic dysfunction, or heart failure in the absence of a prohibitive surgical risk [10,17] and are the same for native valve endocarditis, PVE, and TAVR associated IE [10]. While studies show a clear benefit for surgery for native valve IE and PVE [9,18,19,20], it is currently unclear whether surgical treatment for TAVR-IE patients is beneficial.

There seems to be a certain reluctance to indicate surgery in TAVR-IE patients [15,21,22]. Published data shows that surgical rates of TAVR-IE are only between 2 and 24% [19,23,24,25,26], compared to about 40–50% of all IE patients who were treated surgically [6].

This could be related to the fact that some studies observe no difference in mortality between surgically treated and non-surgically treated TAVR-IE patients. Magner et al. report on an in-hospital mortality of 29.1% in surgically treated patients vs. 32.6% in conservatively treated patients and a 1-year mortality of 47.1% vs. 48.2%, respectively [19]. Bansal et al. report an in-hospital mortality of 9.9% in surgical vs. 12.4% in non-surgical patients [25], and Panagides et al. report on a 1 year mortality of 46.5% in surgical vs. 44.8% in non-surgical patients [27].

Nevertheless, there are studies that were able to show a benefit of surgical therapy for TAVR-IE patients [28,29], especially in patients with local extension of the infection [26]. Saha et al. also reported on similar outcomes in low-intermediate surgical risk patients with TAVR-IE and SAVR-IE treated surgically [14,30].

Notably, previous studies report on data collected before 2020. In our study, we observe a significant increase in cases after 2020 (Figure 1), and over 50% of patients had surgery for TAVR-IE after 2020. Undoubtedly, the trend towards TAVR in patients with lower risk [3,4,5] might have a massive impact on mortality rates if these patients require TAVR-IE surgery. We report on a 1-year mortality rate of 33.6%, which is lower than what Panagides et al. and Mangner et al. reported before [19,27] and in the same range as what Marin-Cuartas reported with 32.8% [15].

In addition, our mortality rate of 18.1% at 30 days is comparable to reports from the EXPLANT-TAVR registry [15] and also comparable to mortality rates reported for PVE surgery [16,31].

These outcomes are comparable to or slightly better than previously reported results for surgically treated TAVR-IE patients, where 30-day mortality ranged from 20% to 35% and 1-year mortality from 30% to 50% [15,19]. They are also in line with contemporary series of surgical prosthetic valve endocarditis (PVE), which typically report early mortality between 15% and 25% and 1-year mortality around 30–40% [16]. Thus, while perioperative mortality remains high, the survival rates achieved in our series suggest that surgical treatment is a viable option in carefully selected patients, particularly when performed in experienced centers.

### 4.2. Causative Microorganism

For microbiological diagnosis of IE, blood cultures remain the cornerstone [10]. Even though it is almost always possible to identify a leading pathogen in the absence of previous antibiotic therapy [10], in our cohort of patients under previous antibiotic therapy, it was only possible to obtain a pathogen result with intraoperative PCR testing in about half of the cases.

Staphylococci, especially Staphylococcus aureus, is the leading pathogen for native and prosthetic valve endocarditis in high-income countries. While Enterocci are the cause of IE in about 10% of all IE patients, TAVR IE patients show a slightly different microbiological spectrum/profile, with Enterocci in the top list of leading pathogens [9,32]. Some studies even identify Enterococci as the most frequent pathogen [23,24,28]. Multiple antibiotic class resistances make treatment with Enterocci difficult [33].

Even though we could not find a difference in mortality between pathogens, Staphyloccus aureus seems to be of high virulence. Del Val et al. report on a doubling of in-hospital and 2-year mortality compared to other pathogens [34]. Ried et al. also report on a 5-fold higher mortality risk with staphylococcal PVE [35]. Accordingly, there are data suggesting that patients with staphylococcal IE should be treated with early surgery to prevent abscess formation [35]. In our data, Staphylococci are the cause for IE in almost half of cases (42%), Staphylococcus aureus alone is the cause in 16% of cases. Enterococci are the cause in 22% of cases.

While previous studies have shown S. aureus to predict poorer outcomes, our cohort did not demonstrate a pathogen–mortality correlation. This discrepancy likely reflects limited sample size and prior antibiotic treatment reducing microbiological yield.

### 4.3. Early and Late IE

Although there are conflicting results in some studies [35,36], there seems to be a similar incidence of IE after TAVR compared to SAVR with an incidence of 0.3–2.0 (per 100 person-years) [9,32]. In addition, Ando et al. report no difference between early (≤12 months) and late (>12 months) IE after TAVR compared to SAVR [37].

In between the group of TAVR patients with IE, multiple studies report more early than late IE [9,27,32]. The question arises whether this circumstance has an impact on outcome.

Nappi et al. [32] report on different microbiological profiles between early and late IE with Staphylococci and increasingly Enterococci as the leading pathogens, while late IE has a similar microbiological profile to that observed in native valve IE.

Our study classifies patients in very early (≤90 days), early (≤1 year), and late IE, according to time between TAVR and surgery for IE, which is not the same as time between TAVR and diagnosis of IE.

We report on a median time between TAVR and TAVR explant of 342 days (11.2 months). This is later than the 7.4 months reported by Mangner et al. [19] and the 10.4 months reported by Marin-Cuartas et al. [15], but earlier than the 17 months reported by Rosch et al. [28] and Saha et al. with a median time between TAVR and surgery of 14 months [30].

Mangner et al. report on a rate of 65% of early IE in their cohort of 111 surgically treated TAVR-IE patients [19], while Saha et al. report on a rate of early IE of 46% [30].

In our cohort, we observed a rate of early IE in 52%, but no differences in median time between TAVR and TAVR explant between our two groups, even though there is a trend towards late IE in the cases group (with a median time of 490 days).

Patients with late IE had a survival rate at 30 days of 73% and a 59% survival rate at 1 year, while patients who were operated on very early post-TAVR had a 30-day survival of 78%, and a 1-year survival rate of only 44%, which was the lowest rate in all subgroups (Figure 6). Interestingly, with 94% at 30 days and 88% at 1 year, we observed the best survival rates for patients who had surgery between 90 days and 1 year after TAVR. This is in accordance with Marin-Cuartas et al., with a report on a higher mortality in patients with late THV explant (>18 months) [15].

Reasons for referring our patients to surgery were in accordance with current guidelines, mainly paravalvular/valvular extensions or abscess formation, but also cases of septic emboli, uncontrolled infection, and valve dysfunction [10,17].

Even though there seems to be some reluctance to refer eligible patients to surgery [15,21,22], with a median hospitalization time of 9 days before surgery, our data does not suggest an overly long pre-operative period.

### 4.4. Periprocedural Aspects

When referring to TAVR-related infective endocarditis (IE), it is mainly associated with isolated infection of the prosthetic valve. Nevertheless, in about half of patients, the infection is not limited to the TAVR prosthesis. In about 15% of cases, the mitral valve and in about 1/3 of patients, at least two cardiac structures are infected [9,19,26].

Our observation confirms this. In our cohort, about half of patients had concomitant surgeries, most commonly on the mitral valve (28%). Marin-Cuartas et al. also report on a similar rate of concomitant surgeries [15]. There was no difference in survival between patients with isolated SAVR and concomitant surgeries. Similarly, we observed no significant difference between our groups. Although there was a tendency toward a higher frequency of concomitant surgeries in the case group, this did not result in a statistically significant difference in operative duration, extracorporeal circulation time, or aortic cross-clamp time.

Patients in the case group had significantly lower left ventricular ejection fraction (LVEF). Impaired LVEF is a well-known predictor of adverse outcomes after cardiac surgery, reflecting reduced myocardial reserve. Our data confirm its prognostic importance in SAVR for IE after TAVR. Ventricular function should be a key factor in Heart Team decision making and may warrant earlier referral to surgery before further deterioration occurs.

Embolic events are common in infective endocarditis and negatively affect prognosis, mainly through neurological and peripheral complications. As shown by Pizzino et al. [38], early recognition and timely intervention are essential to reduce embolic risk. In our cohort, postoperative stroke rates did not differ between groups, but vigilance for embolic complications remains crucial in management.

Postoperatively, we observed no difference in need for a permanent pacemaker or rethoracotomy due to bleeding between our groups. The only perioperative difference was in need for dialysis during ICU stay, with more dialysis for patients in the cases group.

Even though there is no strong evidence that valve type has an impact on IE rates [1,9,37,39,40], Ried et al. observed a 3.4-fold higher risk of PVE on BEV compared to SEV [35]. Zaid et al. also report on fewer cases of IE with SEV [41].

We confirmed these findings, and with 78%, BEVs were by far the most frequent valve types we explanted, with no difference between groups. Ried et al. assume that the pressure to expand the BEV results in more extensive tissue injury, creating microlesions and trauma that could facilitate the development of subsequent IE.

We did not observe significant injury due to prosthesis explantation that would result in aortic root replacement, despite reports of extensive lesions due to removal of the prosthesis [42]. Zaid et al. report on a higher need for aortic root replacement in SEV compared to BEV [41]. All injuries we saw were most likely caused by the infection and were able to be covered with a patch. Anyway, it can be challenging even for experienced surgeons to explant a well grown-in but infected prosthesis of any kind, particularly when other valves are affected as well.

## 5. Conclusions

SAVR for TAVR-associated infective endocarditis carries substantial early risk, yet many survivors achieve functional independence. We hypothesize that with ongoing expansion of TAVR to younger and lower-risk populations, perioperative outcomes may improve; however, this remains speculative and should be validated prospectively. Heterogeneity due to evolving TAVR and surgical technologies during the long study period represents an inherent limitation.

The indication for IE surgery must be concluded on an individual basis. The observed survival rates and the promising clinical outcome demonstrate that even high surgical risk patients have a substantial chance of regaining a self-determined and autonomous life.

Therefore, after careful consideration in the heart team, surgery for TAVR IE can be offered at experienced sites.

## 6. Limitations

It is retrospective observational analysis with all the inherent limitations.

We are limited by our sample size. Also, there might be time selection and learning curve bias due to long time frame between 2008 and 2023. Additionally, we are not able to account for qualifying patients that did not undergo or declined surgery, and we also have no records on patients that had surgery elsewhere. As most procedures were performed in recent years, the median follow up was relatively short despite the high follow-up completeness.

Finally, surgery was performed by different surgeons. The decision to perform additional cardiac procedures was at the surgeon’s discretion at the time of the procedure.

We did not perform standardized functional or quality-of-life assessments; however, according to our institutional policy, discharge home is only considered if patients are deemed capable of managing daily necessities independently, with family support and social service involvement as needed.

## Figures and Tables

**Table 1:** Baseline characteristics.

Variable	All Patients (n = 50)	Cases Group (n = 15)	Control Group (n = 35)	*p* Value
**age, years, median [range]**	78 [37–88]	80 [37–86]	77 [57–88]	0.8
**sex (male), n (%)**	40 (80)	12 (80)	28 (80)	1.0
**height (m)**	1.8 [1.5–1.9]	1.8 [1.5–1.8]	1.8 [1.6–1.9]	0.4
**weight (kg)**	80 [43–150]	80 [60–150]	80 [43–125]	0.7
**body-Mass-Index (kg/m^2^)**	26.7 [16.8–54.7]	27.5 [18.5–54.7]	26.5 [16.8–38.9]	0.5
**white Blood Count (10^9^/L)**	9.42 [3.82–45.58]	9.92 [5.15–45.58]	8.87 [3.82–19.33]	0.12
**c reactive protein (mg/dL)**	69.85 [1.34–365]	75.95 [20.8–365]	64.7 [1.34–265]	0.22
**creatinine (mg/dL)**	1.27 [0.49–10.40]	1.35 [0.49–10.40]	1.23 [0.56–6.16]	0.13
**glomerular filtration rate (mL/min)**	53 [7–144]	47 [7–100]	54 [11–144]	0.053
**cerebrovascular disease, n (%)**	13 (26)	4 (26.7)	9 (25.7)	0.9
**TIA, n (%)**	8 (16)	3 (20)	5 (14.3)	
**stroke, n (%)**	5 (10)	1 (6.7)	4 (11.4)	
**COPD, n (%)**	10 (20)	3 (20)	7 (20)	1.0
**diabetes mellitus**	14 (28)	5 (33.3)	9 (25.7)	0.9
**NIDDM**	6 (12.0%)	2 (13.3%)	4 (11.4%)	
**IDDM**	8 (16.0%)	3 (20.0%)	5 (14.3%)	
**LV-ejection fraction (%)**	47.4 ± 10.4	42.7 ± 8.4	49.4 ± 10.6	0.024
**peripheral arterial disease, n (%)**	5 (10)	2 (13.3)	3 (8.6)	0.6
**previous heart surgery, n (%)**	9 (18)	1 (6.7)	8 (22.9)	0.2
**log Euroscore pre-TAVR,** **median, % [range]**	9.5 [1.3–48.3]	8.1 [1.4–37.6]	10.7 [1.3–48.3]	0.6
**STS-Score pre TAVR, median, % [range]**	2.4 [0.8–5.9]	2.7 [1.5–5.4]	2.1 [0.8–5.9]	0.1
**log Euroscore pre SAVR,** **median, % [range]**	28.5 [6.8–82.1]	53.3 [20.1–82.1]	26.6 [6.8–64.5]	<0.001
**STS PROM pre SAVR, median, % [range]**	6.5 [1.3–67.6]	14.4 [3.9–67.6]	4.8 [1.3–51.2]	0.004
**time between TAVR and** **SAVR, median, days [range]**	342 [35–3248]	490 [35–1634]	328 [74–3248]	0.6
**time between TAVR and SAVR (days)**				0.07
**<90 days, n (%)**	9 (18%)	5 (33.3)	4 (11.4)	
**>90–<364 days, n (%)**	17 (34%)	2 (13.3)	15 (42.9)	
**>365 days, n (%)**	24 (48%)	8 (53.3)	16 (45.7)	

Abbreviations: TIA, transitory ischemic attack; SAVR, surgical aortic valve replacement; CI, confidence interval; range, interquartile range; SD, standard deviation. for categorical variables: n (%), Fisher’s test; for normal distribution: mean ± sd, *t*-test; for non-normal distribution: median [range], Wilcox-Test.

**Table 2:** Causative microorganism.

Causative Microorganism, n (%)	All Patients (n = 50)	Cases Group (n = 15)	Control Group (n = 35)
**Staphylococcus aureus**	8 (16)	4 (26.7)	4 (11.4)
**Coagulase-negative** **staphylococci**	13 (26)	3 (20)	10 (28.6)
**Streptococcus viridans**	8 (16)	0	8 (22.9)
**Enterococci**	11 (22)	4 (26.7)	7 (20)
**others**	6 (12)	3 (20)	3 (8.6)
**no causative microorganism** **found**	3 (6)	1 (6.7)	2 (5.7)

**Table 3:** Periprocedural characteristics.

Periprocedural Characteristics	All Patients (n = 50)	Cases Group (n = 15)	Control Group (n = 35)	*p* Value
**CPB Time, min, median [range]**	137 [54–348]	155 [54–348]	136 [83–316]	0.5
**cross-clamp Time, min,** **median [range]**	95 [36–233]	89 [36–233]	97 [52–220]	0.9
**total operative time, min,** **median [range]**	258 [124–538]	295 [133–538]	255 [124–431]	0.3
**explanted BEV-Device, n (%)**	39 (78)	12 (80)	27 (77.1)	
**explanted SEV-Devices, n (%)**	11 (22)	3 (20)	8 (22.9)	0.12
**concomitant surgery, n (%)**	25 (50.0%)	11 (73.3%)	14 (40.0%)	0.062

Abbreviations: CBP, cardiopulmonary bypass; BEV, balloon-expandable valve; SEV, self-expandable valve; CI, confidence interval; range, interquartile range; SD, standard deviation. for categorical variables: n (%), Fisher’s test; for normal distribution: mean ± sd, *t*-test; for non-normal distribution: median [range], Wilcox’ test.

**Table 4:** In-hospital clinical outcomes.

In-hospital Clinical Outcomes	All Patients (n = 50)	Cases Group (n = 15)	Control Group (n = 35)	*p* Value
**acute renal failure/Dialysis n** **(%)**	22 (44.0%)	13 (86.7%)	9 (25.7%)	<0.001
**Stroke, n (%)**	5 (10)	2 (13.3)	3 (8.6)	0.6
**new pacemaker implantation, n** **(%)**	10 (20)	9 (25.7)	1 (7.1)	0.2
**Rethoracotomy, n (%)**	7 (14)	1 (6.7)	6 (17.1)	0.7
**length of hospital stay, days,** **median [range]**	20 [2–95]	25 [2–42]	19 [3–95]	0.9
**length of ICU stay, days,** **median [range]**	6 [1–95]	23 [1–42]	5 [1–95]	0.07
**VARC3-Mortality,** **n, (%)**				
**alive n, (%)**	30 (60)	0	30 (85.7)	
**periprocedural n,** **(%)**	15 (30)	15 (100)	0	
**early n, (%)**	1 (2)	0	1 (2.9)	
**late n, (%)**	4 (8.0)	0	4 (11.4)	
**estimated 30 day mortality** **(95% CI), %**	81.9 ± 5.5			
**estimated 1-year-mortality** **(95% CI), %**	67.4 ± 6.7			
**follow up (days)**	294 [1–3802]	22 [1–106]	752 [19–3802]	<0.001

Abbreviations: CI, confidence interval; range, interquartile range; SD, standard deviation for categorical variables: n (%), Fisher’s test; for normal distribution: mean ± sd, *t*-test; for non-normal distribution: median [range], Wilcox’ test.

## Data Availability

Data is unavailable due to privacy or ethical restrictions but can be provided anonymously.
